# Chloroplast phylogenomic analysis resolves deep-level relationships within the green algal class Trebouxiophyceae

**DOI:** 10.1186/s12862-014-0211-2

**Published:** 2014-10-01

**Authors:** Claude Lemieux, Christian Otis, Monique Turmel

**Affiliations:** Département de Biochimie, de Microbiologie et de Bio-informatique, Institut de Biologie Intégrative et des Systèmes, Université Laval, 1030 avenue de la Medicine, Pavillon Marchand, G1V 0A6 Québec, Canada

**Keywords:** Chlorophyta, Trebouxiophyceae, Plastid genome, Phylogenomics

## Abstract

**Background:**

The green algae represent one of the most successful groups of photosynthetic eukaryotes, but compared to their land plant relatives, surprisingly little is known about their evolutionary history. This is in great part due to the difficulty of recognizing species diversity behind morphologically similar organisms. The Trebouxiophyceae is a species-rich class of the Chlorophyta that includes symbionts (e.g. lichenized algae) as well as free-living green algae. Members of this group display remarkable ecological variation, occurring in aquatic, terrestrial and aeroterrestrial environments. Because a reliable backbone phylogeny is essential to understand the evolutionary history of the Trebouxiophyceae, we sought to identify the relationships among the major trebouxiophycean lineages that have been previously recognized in nuclear-encoded 18S rRNA phylogenies. To this end, we used a chloroplast phylogenomic approach.

**Results:**

We determined the sequences of 29 chlorophyte chloroplast genomes and assembled amino acid and nucleotide data sets derived from 79 chloroplast genes of 61 chlorophytes, including 35 trebouxiophyceans. The amino acid- and nucleotide-based phylogenies inferred using maximum likelihood and Bayesian methods and various models of sequence evolution revealed essentially the same relationships for the trebouxiophyceans. Two major groups were identified: a strongly supported clade of 29 taxa (core trebouxiophyceans) that is sister to the Chlorophyceae + Ulvophyceae and a clade comprising the Chlorellales and Pedinophyceae that represents a basal divergence relative to the former group. The core trebouxiophyceans form a grade of strongly supported clades that include a novel lineage represented by the desert crust alga *Pleurastrosarcina brevispinosa*. The assemblage composed of the *Oocystis* and *Geminella* clades is the deepest divergence of the core trebouxiophyceans. Like most of the chlorellaleans, early-diverging core trebouxiophyceans are predominantly planktonic species, whereas core trebouxiophyceans occupying more derived lineages are mostly terrestrial or aeroterrestrial algae.

**Conclusions:**

Our phylogenomic study provides a solid foundation for addressing fundamental questions related to the biology and ecology of the Trebouxiophyceae. The inferred trees reveal that this class is not monophyletic; they offer new insights not only into the internal structure of the class but also into the lifestyle of its founding members and subsequent adaptations to changing environments.

## Background

The green algae represent an ancient lineage of photosynthetic eukaryotes; molecular clock analyses estimate their origin between 700 and 1,500 millions years ago [1]. This lineage (Viridiplantae) split very early into two major divisions: the Chlorophyta, containing the majority of the described green algae, and the Streptophyta, containing the charophyte green algae and their land plant descendants. In the last decade, substantial advances have been made in our understanding of the broad-scale relationships among the streptophytes, in particular the land plants [2], and references therein; however, progress has lagged behind concerning the chlorophytes.

Early hypotheses on green algal phylogeny were based on morphology and ultrastructural data derived from the flagellar apparatus and processes of mitosis and cell division [3,4]. These ultrastructural features, which apply to most green algae, supported the existence of the Streptophyta and Chlorophyta and revealed four distinct groups within the Chlorophyta that were recognized as classes: the predominantly marine, unicellular, Prasinophyceae; the predominantly marine and morphologically diverse Ulvophyceae; and the freshwater or terrestrial, morphologically diverse Trebouxiophyceae (=Pleurastrophyceae) and Chlorophyceae [5,6]. It was hypothesized that the Prasinophyceae gave rise to the Ulvophyceae, Trebouxiophyceae and Chlorophyceae (UTC). Later, phylogenetic analyses based on the nuclear-encoded small subunit rRNA gene (18S rDNA) largely corroborated these hypotheses [1,5,7]. It was found, however, that the Prasinophyceae are paraphyletic, with the nine main lineages of prasinophytes identified so far representing the earliest branches of the Chlorophyta [8]. For the Ulvophyceae and Trebouxiophyceae, the limited resolution of 18S rDNA trees made it impossible to assess the monophyly of these classes [1,6,7]. Analyses of 18S rDNA data uncovered a myriad of lineages within each of the three UTC classes, but could not resolve their precise branching order. Despite these uncertainties, many taxonomic revisions have been implemented: new species not distinguished by light microscopy were described, new genera were erected, the circumscription of several main lineages was modified, and existing orders were elevated to the class level (e.g. Chlorodendrophyceae and Pedinophyceae). A recurrent theme that emerged from such studies is the finding that multiple genera containing taxa with reduced morphologies (such as unicells and filaments) are polyphyletic, with members often encompassing more than one class e.g. for Chlorella, [9,10].

For ancient groups of eukaryotes such as the green algae, a large number of genes from many species need to be analyzed using reliable models of sequence evolution to resolve relationships at higher taxonomic levels [11]. Multi-gene data sets can be assembled by concatenating the sequences of protein-coding genes that are shared by the chloroplast or nuclear genomes. The chloroplast phylogenomic studies reported so far for green algae have provided valuable insights into the phylogeny of prasinophytes [12,13], streptophytes [14-18] and the Chlorophyceae [19,20], but only limited information is currently available regarding the relationships within the Trebouxiophyceae. For the Ulvophyceae, an analysis of ten concatenated gene sequences from both the nuclear and chloroplast genomes enabled Cocquyt et al. [21] to resolve the branching pattern of the main lineages of this class. In this context, it is worth mentioning that datasets of concatenated nuclear and chloroplast genes have also proved very useful to reconstruct phylogenetic relationships within specific green algal orders [22].

The present investigation is centered on the Trebouxiophyceae as delineated by Frield [23]. This species-rich class displays remarkable variation in both morphology (comprising unicells, colonies, filaments and blades) and ecology (occurring in diverse terrestrial and aquatic environments) [1,5,7]. No flagellate vegetative form has been identified in this class. Several species (e.g. *Trebouxia, Myrmecia* and *Prasiola*) participate in symbioses with fungi to form lichens [24,25] and others (e.g. *Chlorella*, *Coccomyxa*, and *Elliptochloris*) occur as photosynthetic symbionts in ciliates, metazoa and plants [26]. The Trebouxiophyceae also comprises species that have lost photosynthetic capacity and have evolved free-living or parasitic heterotrophic lifestyles (e.g. *Prototheca* and *Helicosporodium*) [27-29]. Aside from their intrinsic biological interest, trebouxiophycean algae have drawn the attention of the scientific community because of their potential utility in a variety of biotechnological applications such as the production of biofuels or other molecules of high economic value [30,31].

Phylogenies based on 18S rDNA data have identified multiple lineages within the Trebouxiophyceae, and these include the Chlorellales, Trebouxiales, Microthamniales, and the *Prasiola*, *Choricystis*/*Botryococcus*, *Watanabea*, *Oocystis* and *Geminella* clades [32-39]. While the majority of the observed monophyletic groups are composed of several genera, a number of lineages consist of a single species or genus (e.g. *Xylochloris*, *Leptosira, Lobosphaera*). The interrelationships between most of the trebouxiophycean lineages are still unresolved. Interestingly, taxa with highly different morphologies (e.g. the minute unicellular *Stichococcus* and the macroscopic filamentous or blade-shaped *Prasiola*) have been recovered in the same clade, demonstrating that vegetative morphology can evolve relatively rapidly. Polyphyly has been reported not only in morphologically simple genera [5,7,40], but also in those with colonial forms [36,41].

In this study, we have sought to decipher the relationships among the main trebouxiophycean lineages and to evaluate the monophyly of the Trebouxiophyceae. Toward these goals, we have analyzed data sets of 79 chloroplast DNA (cpDNA)-encoded proteins and genes spanning the broad range diversity of the Trebouxiophyceae. Twenty-nine chlorophyte chloroplast genomes were newly sequenced to generate these data sets. The trees we inferred using the maximum likelihood (ML) and Bayesian inference methods enabled us not only to clarify the internal structure of the Trebouxiophyceae but also to gain insights into their ancestral status with regards to the type of environment they first colonized and their subsequent adaptations to different ecosystems.

## Results

In the course of this study, we generated the chloroplast genome sequences of 27 trebouxiophycean taxa, thus bringing to 35 the total number of trebouxiophyceans sampled in our phylogenetic analyses (Table 1). These taxa represent the variety of trebouxiophycean lineages that had been recognized prior to January 2013; at least two representatives were examined for each of the lineages ncluding multiple genera. The chloroplast genome sequences of two flagellates belonging to the Pedinophyceae (*Pedinomonas tuberculata* and *Marsupiomonas* sp. NIES 1824) were also determined because *Pedinomonas minor*, the previously sampled taxon from this group had been found to be related to the Chlorellales and a member of the *Oocystis* lineage in an earlier phylogenomic study [42]. Only the results of our phylogenetic analyses are presented here; in a separate article, we will report the salient features of the newly sequenced chloroplast genomes and discuss how these structural data advance understanding of chloroplast genome evolution in the Chlorophyta.

**Table 1:** **Pedinophycean and trebouxiophycean taxa used in the chloroplast phylogenomic analyses**

**Taxa**	**Source** ^**a**^	**Accession no.** ^**b**^	**Sequencing method** ^**c**^
**Pedinophyceae**			
*Pedinomonas minor*	UTEX LB 1350	[GenBank:NC_016733]	
*Pedinomonas tuberculata*	SAG 42.84	[GenBank:KM462867]*	454
*Marsupiomonas* sp.	NIES 1824	[GenBank:KM462870]*	454
**Trebouxiophyceae**			
**Chlorellales**			
*Pseudochloris wilhelmii*	SAG 1.80	[GenBank:KM462886]*	Illumina
*Chlorella variabilis*	NC64A	[GenBank:NC_015359]	
*Chlorella vulgaris*	C-27	[GenBank:NC_001865]	
*Dicloster acuatus*	SAG 41.98	[GenBank:KM462885]*	Sanger
*Marvania geminata*	SAG 12.88	[GenBank:KM462888]*	454
*Parachlorella kessleri*	SAG 211-11 g	[GenBank:NC_012978]	
***Choricystis/Botryococcus*** **clade**			
*Botryococcus braunii*	SAG 807-1	[GenBank:KM462884]*	Illumina
*Choricystis minor*	SAG 17.98	[GenBank:KM462878]*	Sanger
*Coccomyxa subellipsoidea*	NIES 2166	[GenBank:NC_015084]	
*Elliptochloris bilobata*	CAUP H7103	[GenBank:KM462887]*	454
*Paradoxia multiseta*	SAG 18.84	[GenBank:KM462879]*	Illumina
*Trebouxiophyceae* sp*.*	MX-AZ01	[GenBank:NC_018569]	
***Geminella*** **clade**			
*Geminella minor*	SAG 22.88	[GenBank:KM462883]*	Illumina
*Geminella terricola*	SAG 20.91	[GenBank:KM462881]*	454
*Gloeotilopsis sterilis*	UTEX 1704	[GenBank:KM462877]*	Illumina
**Microthamniales**			
*Fusochloris perforata*	SAG 28.85	[GenBank:KM462882]*	454
*Microthamnion kuetzingianum*	UTEX 318	[GenBank:KM462876]*	Sanger
***Oocystis*** **clade**			
*Oocystis solitaria*	SAG 83.80	[GenBank:FJ968739]	
*Planctonema lauterbornii*	SAG 68.94	[GenBank:KM462880]*	Sanger
***Prasiola*** **clade**			
*“Chlorella” mirabilis*	SAG 38.88	[GenBank:KM462865]*	Sanger
*Koliella longiseta*	UTEX 339	[GenBank:KM462868]*	454
*Pabia signiensis*	SAG 7.90	[GenBank:KM462866]*	Sanger
*Stichococcus bacillaris*	UTEX 176	[GenBank:KM462864]*	Sanger
*Prasiolopsis* sp.	SAG 84.81	[GenBank:KM462862]*	454
**Trebouxiales**			
*Myrmecia israelensis*	UTEX 1181	[GenBank:KM462861]*	454
*Trebouxia aggregata*	SAG 219-1D	[GenBank:EU123962-EU124002]	
***Watanabea*** **clade**			
*Dictyochloropsis reticulata*	SAG 2150	[GenBank:KM462860]*	454
*Watanabea reniformis*	SAG 211-9b	[GenBank:KM462863]*	Illumina
**Other lineages**			
*Pleurastrosarcina brevispinosa* ^d^	UTEX 1176	[GenBank:KM462875]*	Illumina
*“Koliella” corcontica*	SAG 24.84	[GenBank:KM462874]*	Illumina
*Leptosira terrestris*	UTEX 333	[GenBank:NC_009681]	
*Lobosphaera incisa*	SAG 2007	[GenBank:KM462871]*	Sanger
*Neocystis brevis*	CAUP D802	[GenBank:KM462873]*	454
*Parietochloris pseudoalveolaris*	UTEX 975	[GenBank:KM462869]*	Sanger
*Xylochloris irregularis*	CAUP H7801	[GenBank:KM462872]*	454

^a^The taxa originate from the culture collections of algae at the University of Goettingen (SAG, [43]), the University of Texas at Austin (UTEX, [44]), the Provasoli-Guillard National Center for Marine Algae and Microbiota (CCMP, [45]), the National Institute of Environmental Studies in Tsukuba (NIES, [46]), and Charles University in Prague (CAUP, [47]).

^b^The GenBank accession number of the chloroplast genome is given for each taxon. The asterisks denote the genomes that were sequenced during the course of this study.

^c^Sequencing methods are given only for the chloroplast genomes sequenced in this study.

^d^This taxon, originally classified in the genus *Chlorosarcina*, was assigned to the new genus *Pleurastrosarcina* by Sluiman and Blommers [48].

**Figure 1:**
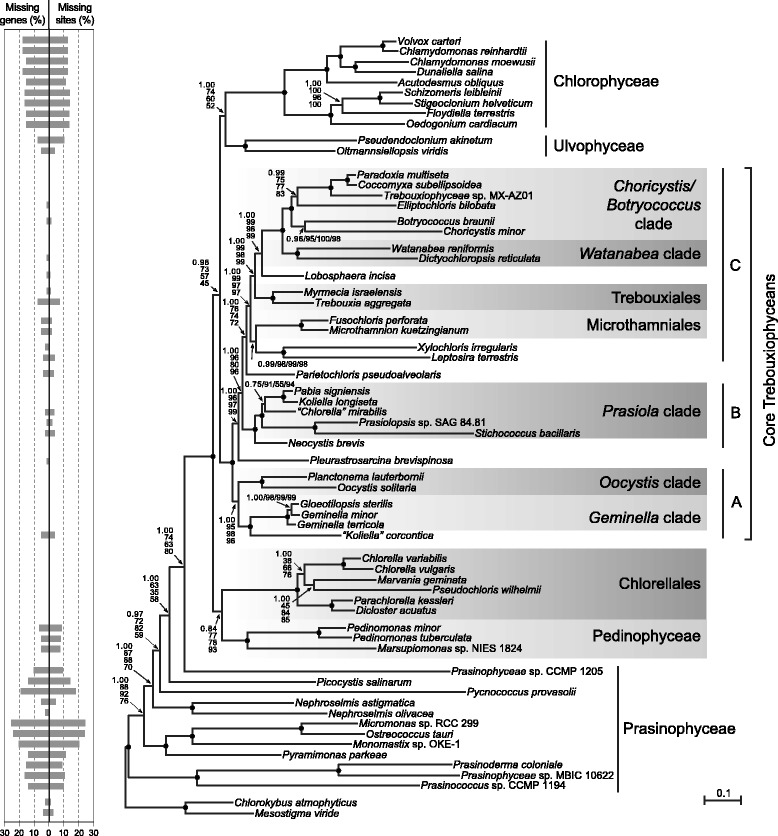
**Phylogeny of 61 chlorophytes inferred using a data set of 15,549 positions assembled from 79 cpDNA-encoded proteins.** The tree presented here is the best-scoring ML tree inferred under the GTR + Γ4 model. Support values are reported on the nodes: from top to bottom, or from left to right, are shown the posterior probability (PP) values for the PhyloBayes CATGTR + Γ4 analyses and the bootstrap support (BS) values for the RAxML GTR + Γ4, LG4X and gcpREV + Γ4 analyses. Black dots indicate that the corresponding branches received BS and PP values of 100% in all four analyses. Shaded areas identify the clades that are well supported in 18S rDNA phylogenies. The histograms on the left indicate the proportion of missing data for each taxon. The scale bar denotes the estimated number of amino acid substitutions per site.

All data sets analyzed in our study were assembled from 79 cpDNA-encoded proteins and taxon sampling included up to 63 green algal taxa, i.e. the 38 trebouxiophyceans and pedinophyceans listed in Table 1, 23 additional chlorophytes (12 prasinophytes, nine chlorophyceans, and two ulvophyceans) and two streptophyte algae (*Mesostigma viride* and *Chlorokybus atmophyticus*). We favored the use of amino acid rather than nucleotide sequences in our phylogenomic study because, in analyses of ancient divergences, amino acid data sets are less prone than nucleotide data sets to saturation problems, convergent compositional biases and convergent codon-usage biases [49-51]. We initiated our phylogenomic study by analyzing the amino acid data set comprising all 63 taxa (15,549 sites). Note that some of the genes coding for the proteins analyzed are missing from a number of taxa, in particular from prasinophytes and chlorophyceans (see Figure 1); however, the proportion of missing data in the analyzed data sets does not exceed 6%.

Even though amino acid phylogenies are more robust to compositional effects than nucleotide phylogenies, they may still suffer from a general mutational pressure acting at the nucleotide level [52,53]. For this reason, we also inferred trees from nucleotide data sets corresponding to the 63-taxon amino acid data set and examined whether they are congruent with those derived from amino acid data sets.

### Analysis of the amino acid data sets

The amino acid data set comprising all 63 taxa was analyzed with PhyloBayes using the site-heterogeneous CATGTR + Γ4 model and also with RAxML using the site-homogeneous GTR + Γ4 and gcpREV + Γ4 models as well as the LG4X model (Figure 1). gcpREV is an empirical amino acid substitution model that has been recently developed for use with green plant chloroplast protein data [54]; it proved to be the best-scoring empirical model among those we tested using RAxML (cpREV, JTT, gcpREV, LG, WAG, and their + F alternatives). LG4X is a mixture model based on four substitution matrices [55]. The fits of the gcpREV + Γ4, GTR + Γ4 and CATGTR + Γ4 models to the 63-taxon data set were assessed using cross-validation (Table 2). CATGTR + Γ4 was found to be the best-fitting model; this finding was expected considering that site-heterogeneous models are known to provide a better fit than site-homogeneous models and minimize the impact of systematic errors arising from the difficulties to detect and interpret multiple substitutions [56-59]. Because it was also found that the GTR + Γ4 model has a better fit than the gcpREV + Γ4 model (Table 2), it appears that the size of the 63-taxon data set is sufficiently large to estimate a GTR amino acid substitution matrix that models more accurately our data than the empirical gcpREV matrix.

**Table 2:** **Comparison of evolutionary models using cross validation and the chloroplast data set of 15,549 positions**

**Models compared**	**Likelihood difference (±SD)**
GTR + Γ4 vs gcpREV + Γ4	396.52 ± 48.58
CATGTR + Γ4 vs gcpREV + Γ4	2545.78 ± 133.97

The majority-rule consensus trees inferred from the 63-taxon amino acid data set using ML and Bayesian inference methods displayed essentially the same topology (Figure 1). As expected, the prasinophyte lineages represent the first branches and their divergence order is identical to that reported for a recent phylogenomic tree with the same sampling of prasinophyte taxa [12]. The trebouxiophyceans are recovered as a non-monophyletic assemblage. The monophyletic group formed by the six members of the Chlorellales is sister to the Pedinophyceae and the Chlorellales + Pedinophyceae clade is sister to all other UTC algae. The rest of the trebouxiophyceans, designated hereafter as core trebouxiophyceans, form a strongly supported clade that shares a sister relationship with the Ulvophyceae + Chlorophyceae clade. The deep node of the trees coinciding with the common ancestor of the UTC and pedinophycean algae received maximal support in all analyses, but the following node corresponding to the divergence of the core trebouxiophyceans from the Chlorellales + Pedinophyceae received lower support, especially in the ML analyses as indicated by the BS values of 73, 57 and 45%.

The 32 taxa within the core trebouxiophyceans are resolved as a grade of several strongly supported lineages. Three monophyletic groups containing multiple genera can be distinguished (i.e. clades A, B and C). Clade A, which consists of *Koliella corcontica* and members of the previously recognized *Geminella* and *Oocystis* clades, represents the earliest-diverging lineage of the core trebouxiophyceans. Clade B includes *Neocystis brevis* and representatives of the highly diversified *Prasiola* clade. Clade C, the largest of the three identified monophyletic groups, consists of 15 taxa belonging to the *Xylochloris,* Microthamniales, Trebouxiales*, Lobosphaera, Watanabea, Choricystis* and *Elliptochloris* clades. Clades A and B as well as clades B and C are separated from one another by a lineage consisting of a single taxon, i.e. the *Pleurastrosarcina brevispinosa* and the *Parietochloris pseudoalveolaris* lineage, respectively.

**Figure 2:**
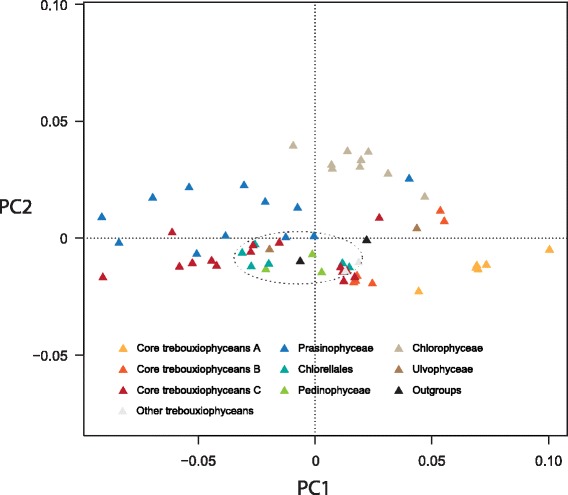
**Correspondence analysis of amino acid usage in the data set of 15,549 positions.** The members of the Chlorellales and Pedinophyceae are found within the circled area.

Considering that heterogeneity in amino acid composition may violate the stationarity assumption made by the evolutionary models in the analyses presented above, we explored whether the inferred relationships were affected by compositional-related artifacts. As a first approach, we examined the amino acid composition of the data set by plotting the first two components of a correspondence analysis of the 20 amino acid frequencies (Figure 2) but identified no large deviation in composition of the chloroplast proteins among the taxa examined. We also used the Dayhoff recoding strategy, which recodes the 20 amino acids into six groups on the basis of their physical and chemical properties. We found that the tree inferred from the Dayhoff-recoded data set under the CATGTR + Γ4 model exhibits the same topology as that obtained using standard 20 state models, except that the Chlorellales are not affiliated with the Pedinophyceae (data not shown). In this Bayesian analysis, which showed convergence problems (maxdiff = 1), the position of the Chlorellales relative to the core trebouxiophyceans is unresolved, whereas the Pedinophyceae is sister to the UTC clade (PP = 0.79). These observations together with the finding that the Chlorellales and Pedinophyceae are grouped in the correspondence analysis (Figure 2) suggest a possible compositional attraction between these two groups.

**Figure 3:**
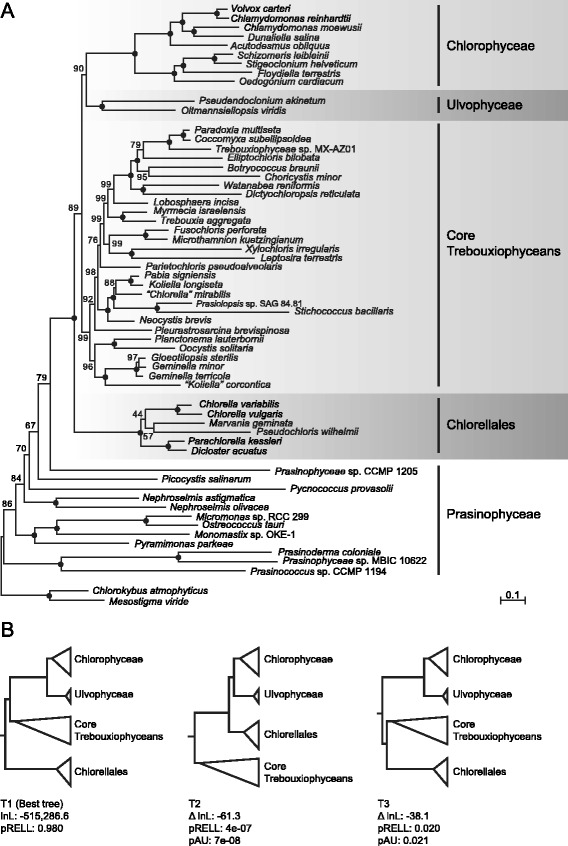
**Influence of the Pedinophyceae on the placement of the Chlorellales. (A)** Phylogeny of chlorophytes inferred under the GTR + Γ4 model using the amino acid data set of 15,549 positions after exclusion of the Pedinophyceae. The best-scoring RAxML tree is presented and support values are reported on the nodes, with black dots indicating 100% BS values. The scale bar denotes the estimated number of amino acid substitutions per site. **(B)** Confidence assessment of the three possible topologies (T1, T2 and T3) for the placement of the Chlorellales under the GTR + Γ4 model. The ΔlnL value indicates the difference in log likelihood relative to the best tree (T1). Local bootstrap probabilities were estimated by resampling of the estimated log-likelihood (RELL). pAU, *P* value for the approximately unbiased (AU) test [60] as implemented in CONSEL 0.20 [61].

Given the possibility that the affiliation between the Chlorellales and Pedinophyceae is caused by systematic errors of tree reconstruction, we tested whether removal of the three members of the Pedinophyceae affects the position of the Chlorellales. As shown in Figure 3A, the RAxML tree inferred under the GTR + Γ4 model still identifies the Chlorellales as sister to the Chlorophyceae + Ulvophyceae + core trebouxiophyceans (BS = 89%). To determine whether the two other possible positions occupied by the Chlorellales (topologies T2 and T3 in Figure 3B) can be dismissed with statistical confidence, we carried out the approximately unbiased (AU) test of phylogenetic tree selection [60]. Both topologies were found to be significantly different (*P* <0.05) from the best tree (T1) and were thus rejected by the AU test (Figure 3B).

### Analysis of the nucleotide data sets

**Figure 4:**
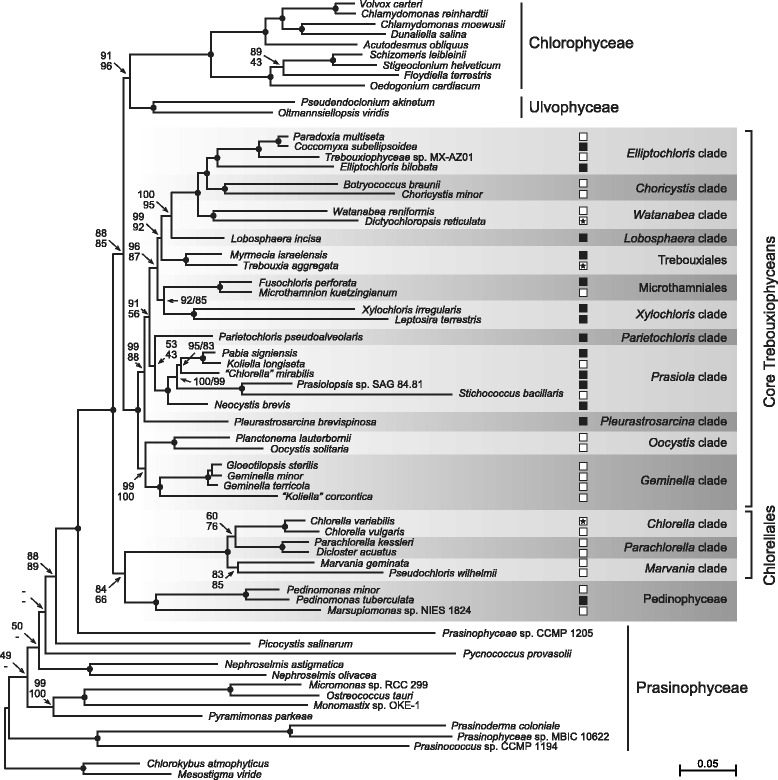
**Phylogeny of 61 chlorophytes inferred using nucleotide data sets assembled from 79 cpDNA-encoded genes.** The tree presented here is the best-scoring ML tree inferred using the degen1 data set under the GTR + Γ4 model. Support values are reported on the nodes: from top to bottom, or from left to right, are shown the BS values for the analyses of the degen1 and nt1 + 2 data sets. Black dots indicate that the corresponding branches received BS values of 100% in the two analyses. Shaded areas identify the trebouxiophycean lineages uncovered in this study. Open and filled squares denote aquatic and terrestrial/aeroterrestrial habitats, respectively; an open square containing a star indicates that the taxon is a symbiont. The scale bar denotes the estimated number of nucleotide substitutions per site.

We analyzed two nucleotide data sets corresponding to the 63-taxon amino acid data set, both of which were designed to minimize deleterious effects of rapid sequence evolution and/or heterogeneous composition. The degen1 data set comprises all three codon positions (46,404 sites) that were degenerated using the Degen1.pl script [62], whereas the nt1 + 2 data set contains only the first and second codon positions (30,936 sites). The RAxML trees inferred from these data sets under the GTR + Γ4 model display essentially the same trebouxiophycean relationships as in the 63-taxon amino acid tree (Figure 4), except that the *Marvania* clade is sister to the *Chlorella* + *Parachlorella* clade (BS = 60 and 76%) and that *Parietochloris pseudoalveolaris* is recovered as sister to the *Prasiola* clade (BS = 53 and 43%). As observed for the amino acid phylogenies, the Chlorellales remained sister to the Chlorophyceae + Ulvophyceae + core trebouxiophyceans when the three algae belonging to the Pedinophyceae were excluded from the sampled taxa (data not shown).

## Discussion

Identifying the relationships among the main lineages of the Trebouxiophyceae is crucial for understanding the evolutionary history of this morphologically and ecologically diversified class of chlorophytes. For the first time, a robust phylogeny of trebouxiophyceans with sampling of most of the lineages recognized on the basis of 18S rDNA data is inferred using a phylogenomic approach. Our study reveals that the class Trebouxiophyceae sensu stricto [23] is not a monophyletic group. In the chloroplast phylogenies we inferred from both amino acid and nucleotide data sets, the Chlorellales and a core group containing all other 29 trebouxiophyceans constitute two distinct, strongly supported monophyletic groups that emerge before the Chlorophyceae and Ulvophyceae (Figures 1 and 4). Prior to our investigation, a number of multi-gene trees with sparse sampling of trebouxiophyceans had recovered with little support the Trebouxiophyceae as nonmonophyletic [2,42,63-67], thus casting doubt on the monophyletic status of this class.

To our knowledge, no morphological features can be invoked to support or refute the phylogenetic relationship we observed between the Chlorellales and the core trebouxiophyceans. Mattox and Stewart [3] defined the class Pleurastrophyceae (=Trebouxiophyceae) based on the ultrastructure of the flagellar apparatus (counterclockwise orientation of basal bodies) and features related to cytokinesis and mitosis (phycoplast-mediated cytokinesis and mitosis with a non-persistent telophase spindle). Because all members of the Chlorellales lack motile stages and divide by autosporulation, the ultrastructural characters used by Mattox and Stewart are not available for this algal group, thus precluding an evaluation of the monophyletic status of the Trebouxiophyceae sensu stricto [23].

The phylogenetic relationships inferred in this study provide insights into the type of ecosystems colonized by the core trebouxiophyceans in their early evolutionary history (Figure 4). Considering that, like most of the chlorellaleans, the earliest-diverging core trebouxiophyceans (i.e. the *Oocystis* and *Geminella* clades) are predominantly planktonic species and that the core trebouxiophyceans occupying more derived lineages are mostly terrestrial algae, it appears that the first core trebouxiophyceans lived in aquatic ecosystems and that very early during evolution they evolved strategies to avoid desiccation [68] and conquered the land. This early transition from aquatic to terrestrial environments likely occurred just after the emergence of the *Oocystis*/*Geminella* clade. In this context, it is worth mentioning that a subaerial lifestyle has been inferred for the last common ancestor of the early-diverging clade *Prasiola*, which comprises terrestrial as well as aquatic species [69]. Therefore, the early evolution of desiccation tolerance undoubtedly accounts for the success of the core trebouxiophyceans in terrestrial/aeroterrestrial environments, and once this trait was acquired, reversals to aquatic habitats probably involved only minor molecular changes, explaining why transitions from terrestrial to aquatic habitats were frequent during the evolution of core trebouxiophyceans.

### The main lineages of the core trebouxiophyceans

The core trebouxiophyceans form a grade of lineages, with several containing two or more genera and some containing a single known genus or taxon. Although the short internal branches separating the major clades of core trebouxiophyceans suggest that lineage diversification occurred rapidly, it is remarkable that only the placement of the single-taxon lineage occupied by the terrestrial alga *Parietochloris pseudoalveolaris* is supported by modest BS values in both the amino acid and nucleotide analyses (Figures 1 and 4). We highlight below the main evolutionary relationships uncovered for the core trebouxiophyceans in our chloroplast phylogenomic study.

The strongly supported assemblage formed by the *Oocystis* and *Geminella* clades represents the deepest branching trebouxiophycean lineage in both the protein- and DNA-based phylogenies (Figures 1 and 4). The placement of the *Oocystis* clade within the core trebouxiophyceans contrasts sharply with the sister relationship of the Oocystaceae and Chlorellales observed in a number of 18S rDNA studies [32,37-39,70]. With regards to the *Geminella* clade, we found that the “*Koliella*” *corcontica* taxon is robustly allied with this clade and thus should be considered to be a *bona fide* member; this association was previously observed in a phylogeny inferred from 18S rDNA, albeit with no support [37].

The sarcinoid green alga *Pleurastrosarcina brevispinosa*, for which no 18S rDNA sequence is currently available in public databases, occupies the next branch after the *Oocystis*/*Geminella* lineages. This desert crust alga, originally designated as *Chlorosarcina brevispinosa*, was assigned to the genus *Pleurastrosarcina* by Sluiman and Blommers [48]. The phylogenies reported here confirm that this taxon belongs to the Trebouxiophyceae and indicate that it represents a novel lineage of this class. In a very recent study, Fučíková et al. [71] reported that most major trebouxiophycean lineages contain desert-dwelling taxa and presented evidence for three new lineages of free-living trebouxiophyceans found in North American desert soil crusts. While the *Desertella* lineage is nested within the *Watanabea* clade, the *Eremochloris* and *Xerochlorella* lineages represent independent clades of the Trebouxiophyceae. In future studies, it will be interesting to investigate whether the sarcinoid *Pleurastrosarcina brevispinosa* belongs to one of the latter lineages. Another lineage that should examined for a possible affinity with *Pleurastrosarcina* is the *Leptochlorella* clade, which was recently discovered by Neustupa et al. [38] and further delineated by Fučíková et al. [71].

The branching order observed for the representatives of the *Prasiola* clade is mostly congruent with 18S rDNA phylogenies [33-35,39], and in agreement with the studies of Krienitz et al. [72] and Gaysina et al. [70], the crescent-shaped green alga *Neocystis brevis* is recovered as sister to this clade. Given that this affiliation is supported with maximal BS values in all analyses, the *Neocystis* lineage clearly represents a basal branch of the *Prasiola* clade. *Chlorella mirabilis* shares a sister relationship with the *Pabia* + *Koliella* clade in all our analyses (Figures 1 and 4); in contrast, 18S rDNA trees frequently identify *C. mirabilis* as sister to all other lineages of the *Prasiola* clade [32-35,39].

The coccoid soil alga *Parietochloris pseudoalveolaris* forms an independent lineage between the *Prasiola* clade and the monophyletic group uniting the Microthamniales and the *Xylochloris* clade in the amino acid-based phylogeny (Figure 1). *Parietochloris* is allied with the Microthamniales in a number of published 18S rDNA trees [32-34,37,38,73], but this alliance is weakly supported. The *Xylochloris* clade is a newly identified assemblage of two lineages for which no sister groups were previously identified; it consists of the coccoid subaerial alga *Xylochloris irregularis* and the filamentatous soil alga *Leptosira terrestris*. The recent discovery of a coccoid soil alga (*Chloropyrula uraliensis*) belonging to a lineage related to the genus *Leptosira* suggests that the *Xylochloris* clade likely represents a diversified group of trebouxiophyceans [70].

The five remaining clades of core trebouxiophyceans consist of the Trebouxiales and the *Lobosphaera*, *Watanabea*, *Choricystis* and *Elliptochloris* clades. Members of all these clades, except the *Lobosphaera* lineage, include algae that occur as symbionts; the Trebouxiales, in particular, are the most common photobionts in lichens. The branching order reported here for the five clades of core trebouxiophyceans was not observed in 18S rDNA trees, even though these clades were often found as neighboring lineages. Only the most recent divergence of core trebouxiophycean lineages we identified (i.e. the *Choricystis*/*Elliptochloris* + *Watanabea* assemblage) was also recivered in 18S rDNA studies [32,72], but with no support. In contrast to 18S rDNA trees where the Trebouxiales and the *Lobosphaera* clade display an unsupported sister relationship [32,33,38,72], the *Lobosphaera* clade consistently emerges with strong support as an independent lineage after the Trebouxiales in all chloroplast trees.

### The Chlorellales and their relationship with other core chlorophytes

Three distinct clades of Chlorellales were recovered in this study: the *Parachlorella*, *Chlorella* and *Marvania* clades (Figure 4). As observed by Somogyi et al. [74] in 18S rDNA trees (albeit with no support), we found that the *Parachlorella* clade is sister to the other two lineages in most amino acid-based trees; however, this position is occupied by the *Marvania* clade in the phylogenies inferred from nucleotide data. A recent 18S rDNA study [75] recovered *Pseudochloris wilhelmii* and the *Parachlorella* and *Chlorella* clades as part of a large assemblage that is sister to *Marvania*, a topology that contrasts with the finding that *Marvania* and *Pseudochloris* are sister taxa in all our analyses.

The results presented here reveal an affinity between the Chlorellales and the Pedinophyceae, although support is weak in the Bayesian analysis under the CATGTR + Γ4 model (PP = 0.84, Figure 1). This finding is consistent with previous chloroplast phylogenomic studies with scarce sampling of trebouxiophyceans, wherein the freshwater flagellate *Pedinomonas minor* was found to be sister to the clade formed by members of the Chlorellales [42,66]. But subsequently, Marin [76] identified no association between the Pedinophyceae and the Chlorellales using nuclear and chloroplast rRNA operon data sets, the Pedinophyceae being placed as an independent lineage that is sister to the Chlorodendrophyceae + UTC. Note that the clade formed by the Chlorellales and other trebouxiophyceans was not supported with high confidence in these rRNA operon trees and that the branching order of most trebouxiophycean lineages was unresolved.

Given the conflicting positions of the Chlorellales and Pedinophyceae in the aforementioned analyses, the weak PP support that the Chlorellales + Pedinophyceae clade received in the PhyloBayes analyses of the amino acid data set and the basal position occupied by the Pedinophyceae in trees inferred from the Dayhoff-recoded data set, we conclude that the question as to whether the Chlorellales and Pedinophyceae form a monophyletic group remains unsettled. It is possible that the Chlorellales + Pedinophyceae affiliation is the result of systematic errors of phylogenetic reconstructions. Solving this issue will require sampling of the Chlorodendrophyceae and the inclusion of additional taxa from the Ulvophyceae and the lineage represented by the prasinophyte CCMP 1205. The two ulvophycean taxa used in our study represent distinct basal lineages of the Ulvophyceae (Oltmannsiellopsidales and Ulvales/Ulotrichales); taxa from the BCDT (Bryopsidales, Cladophorales, Dasycladales, and Trentepohliales) and *Ignatius* clades will need to be examined for a more representative sampling of ulvophycean diversity [21,65]. We expect that resolving the ancient and rapid radiations of the core chlorophyte lineages (Pedinophyceae, Chlorodendrophyceae and UTC lineages) using a chloroplast phylogenomic approach will be challenging and will require optimized models of sequence evolution.

## Conclusions

The phylogeny reported in this study forms a solid basis for future studies aimed at advancing knowledge about the nature of the morphological and ecological diversification of the Trebouxiophyceae. It provides important insights into the origins and adaptations of terrestrial and symbiotic lifestyles. Members of this group clearly occupy a pivotal position in the Viridiplantae and display considerable genetic diversity. A fundamental understanding of the molecular mechanisms underlying their adaptations to changing environments will require the analysis of genomes from key trebouxiophycean taxa.

## Methods

### Strains and culture conditions

The 29 green algal strains that were selected for chloroplast genome sequencing are listed in Table 1 (those are the strains whose accession number is associated with an asterisk). All strains were grown in K [77] or C [78] medium at 18°C under alternating 12 h-light/12 h-dark periods.

### Genome sequencing, assembly and annotation

As indicated in Table 1, three methods were used to determine the sequences of the 29 green algal chloroplast genomes. Nine of these genomes were sequenced using the Sanger method, 12 using the 454 pyrosequencing method, and the remaining eight using the Illumina method. Sanger sequencing was carried out from random clone libraries of A + T-rich DNA fractions as described [79]. Chloroplast genome sequences were assembled using Sequencher 5.1 (Gene Codes Corporation, Ann Arbor, MI) and genomic regions not represented in the assemblies were sequenced from polymerase chain reaction (PCR)-amplified fragments using primers specific to the flanking contigs.

For 454 sequencing, shotgun libraries of A + T-rich DNA fractions (700-bp fragments) were constructed using the GS-FLX Titanium Rapid Library Preparation Kit of Roche 454 Life Sciences (Branford, CT, USA). Library construction and 454 GS-FLX DNA Titanium pyrosequencing were carried out by the “Plateforme d’Analyses Génomiques de l’Université Laval” [80]. Reads were assembled using Newbler v2.5 [81] with default parameters, and contigs were visualized, linked and edited using the CONSED 22 package [82]. Contigs of chloroplast origin were identified by BLAST searches against a local database of organelle genomes. Regions spanning gaps in the chloroplast assemblies were amplified by PCR with primers specific to the flanking sequences. Purified PCR products were sequenced using Sanger chemistry with the PRISM BigDye Terminator Ready Reaction Cycle Sequencing Kit (Applied Biosystems, Foster City, CA, USA).

For Illumina sequencing, total cellular DNA was isolated using the EZNA HP Plant Mini Kit of Omega Bio-Tek (Norcross, GA, USA). Libraries of 700-bp fragments were constructed using the TrueSeq DNA Sample Prep Kit (Illumina, San Diego, CA, USA) and paired-end reads were generated on the Illumina HiSeq 2000 (100-bp reads) or the MiSeq (300-bp reads) sequencing platforms by the Innovation Centre of McGill University and Genome Quebec [83] and the “Plateforme d’Analyses Génomiques de l’Université Laval” [80], respectively. Reads were assembled using Ray 2.3.1 [84] and contigs were visualized, linked and edited using the CONSED 22 package [82]. Identification of chloroplast contigs and gap filling were performed as described above for 454 sequence assemblies.

Genes and ORFs were identified on the final assemblies using a custom-built suite of bioinformatics tools [85]. Genes coding for rRNAs and tRNAs were localized using RNAmmer [86] and tRNAscan-SE [87], respectively. Intron boundaries were determined by modeling intron secondary structures [88,89] and by comparing intron-containing genes with intronless homologs.

### Phylogenomic analyses of amino acid data sets

The chloroplast genomes of 63 green algal taxa were used in the phylogenomic analyses. The GenBank accession numbers of the pedinophycean and trebouxiophycean genomes are presented in Table 1; those of the remaining taxa are as follows: *Mesostigma viride*, [GenBank:NC_002186]; *Chlorokybus atmophyticus*, [GenBank:NC_008822]; *Prasinococcus* sp. CCMP 1194, [GenBank:KJ746597]; *Prasinoderma coloniale* CCMP 1220, [GenBank:KJ746598]; Prasinophyceae sp. MBIC 106222, [GenBank:KJ746602]; *Pyramimonas parkeae*, [GenBank:NC_012099]; *Monomastix* sp. OKE-1, [GenBank:NC_012101]; *Ostreococcus tauri*, [GenBank:NC_008289]; *Micromonas* sp. RCC 299, [GenBank:NC_012575]; *Nephroselmis olivacea*, [GenBank:NC_000927]; *Nephroselmis astigmatica*, [GenBank:KJ746600]; *Pycnococcus provasolii*, [GenBank:NC_012097]; *Picocystis salinarum*, [GenBank:KJ746599]; Prasinophyceae sp. CCMP 1205, [GenBank:KJ746601]; *Oltmannsiellopsis viridis*, [GenBank:NC_008099]; *Pseudendoclonium akinetum*, [GenBank:NC_008114]; *Oedogonium cardiacum*, [GenBank:NC_011031]; *Floydiella terrestris*, [GenBank:NC_014346]; *Stigeoclonium helveticum*, [GenBank:NC_008372]; *Schizomeris leibleinii*, [GenBank:NC_015645]; *Scenedesmus obliquus*, [GenBank:NC_008101]; *Chlamydomonas moewusii*, [GenBank:EF587443-EF587503]; *Dunaliella salina*, [GenBank:NC_016732]; *Volvox carteri* f. *nagariensis*, [GenBank:GU084820]; and *Chlamydomonas reinhardtii*, [GenBank:NC_005353].

A total of 79 protein-coding genes were used to construct the data sets: *accD, atpA, B, E, F, H, I, ccsA, cemA, chlB, I, L, N, clpP, cysA, T, ftsH, infA, minD, petA, B, D, G, L, psaA, B, C, I, J, M, psbA, B, C, D, E, F, H, I, J, K, L, M, N, T, Z, rbcL, rpl2, 5, 12, 14, 16, 19, 20, 23, 32, 36, rpoA, B, C1, C2, rps2, 3, 4, 7, 8, 9, 11, 12, 14, 18, 19, tufA, ycf1, 3, 4, 12, 20, 47, 62.* Amino acid data sets were prepared as follows: the deduced amino acid sequences from the 79 individual genes were aligned using MUSCLE 3.7 [90], the ambiguously aligned regions in each alignment were removed using TRIMAL 1.3 [91] with the options block = 6, gt = 0.7, st = 0.005 and sw = 3, and the protein alignments were concatenated using Phyutility 2.2.6 [92].

Phylogenies were inferred from the amino acid data sets using the ML and Bayesian methods. ML analyses were carried out using RAxML 8.0.20 [93] and the gcpREV + Γ4 [54], LG4X [55] and GTR + Γ4 models of sequence evolution; in these analyses, the data sets were partitioned by gene, with the model applied to each partition. Confidence of branch points was estimated by fast-bootstrap analysis (f = a) with 500 replicates and confidence assessment of phylogenetic tree selections under the GTR + Γ4 model was carried out by the approximately unbiased (AU) test [60] as implemented in CONSEL 0.20 [61]. Bayesian analyses were performed with PhyloBayes 3.3f [94] using the site-heterogeneous CATGTR + Γ4 model [57]. To establish the appropriate conditions for these analyses, five independent chains were run for 2,000 cycles and consensus topologies were calculated from the saved trees using the BPCOMP program of PhyloBayes after a burn-in of 500 cycles. Under these conditions, the largest discrepancy observed across all bipartitions in the consensus topologies (maxdiff) was lower than 0.30, indicating that convergence between the chains was achieved. Bayesian analysis of the Dayhoff-recoded version of the amino acid data set was also performed using PhyloBayes and the CATGTR + Γ4 model.

Cross-validation tests were conducted to evaluate the fits of the gcpREV + Γ4, GTR + Γ4 and CATGTR + Γ4 models of amino acid substitutions to the data set. They were carried out with PhyloBayes using ten randomly generated replicates. Cross-validation is a very general statistical method for comparing models. The procedure can be summarized as follows. The data set is randomly partitioned into two unequal subsets, the learning set (also called the training set) and the test set. The learning set serves to estimate the parameters of the model and these parameters are then used to compute the likelihood of the test set. To reduce variability, multiple rounds of cross-validation are performed using different partitions and the resulting log likelihood scores (which measure how well the test sets were predicted by the model) are averaged over the rounds.

To analyze the amino acid composition of the 63-taxon data set, we first assembled a 20 × 63 matrix containing the frequency of each amino acid per species using the program Pepstats of the EMBOSS package [95]. A correspondence analysis of this data set was then performed using the R package *ca* [96].

### Phylogenomic analyses of nucleotide data sets

Nucleotide data sets containing the gene sequences represented in the amino acid data set of 63 taxa were prepared as follows. To obtain the data set with all three codon positions, the multiple sequence alignment of each protein was converted into a codon alignment, the poorly aligned and divergent regions in each codon alignment were excluded using Gblocks 0.91b [97] with the -t = c, −b3 = 5, −b4 = 5 and -b5 = half options, and the individual codon alignments were concatenated using Phyutility 2.2.6 [92]. The nt1 + 2 data set was obtained by excluding the third codon positions using Mesquite 2.75 [98]. The degen1 data set was prepared using the Degen1.pl 1.2 script of Regier et al. [62]. This script fully degenerates all codons that encode single amino acids by substituting one of the four standard nucleotides with ambiguity codes that allow for all possible synonymous change for that amino acid. It operates by degenerating nucleotides at all sites that can potentially undergo synonymous change in all pairwise comparisons of sequences in the data matrix, thereby making synonymous change largely invisible and reducing compositional heterogeneity but leaving the inference of nonsynonymous changes largely intact.

ML analyses of nucleotide data sets were carried out using RAxML 8.0.20 [93] and the GTR + Γ4 model of sequence evolution; in these analyses, the data sets were partitioned by gene, with the model applied to each partition. Confidence of branch points was estimated by fast-bootstrap analysis (f = a) with 500 replicates.

### Availability of supporting data

The sequence data generated in this study are available in GenBank under the accession numbers KM462860-KM462888 (see Table 1). The data sets supporting the results of this article are available in the Dryad Digital Repository (doi: 10.5061/dryad.q4432) [99].

## Abbreviations

BS: Bootstrap support
cpDNA: Chloroplast DNA
PP: Posterior probability
UTC: Ulvophyceae, Trebouxiophyceae and Chlorophyceae

## References

[1] Leliaert F, Smith DR, Moreau H, Herron MD, Verbruggen H, Delwiche CF, De Clerck O (2012). Phylogeny and molecular evolution of the green algae. CRC Crit Rev Plant Sci.

[2] Ruhfel BR, Gitzendanner MA, Soltis PS, Soltis DE, Burleigh JG (2014). From algae to angiosperms-inferring the phylogeny of green plants (*Viridiplantae*) from 360 plastid genomes. BMC Evol Biol.

[3] Mattox KR, Stewart KD, Irvine DEG, John DM (1984). Classification of the Green Algae: A Concept Based on Comparative Cytology. The Systematics of the Green Algae.

[4] O’Kelly CJ, Floyd GL (1984). Flagellar apparatus absolute orientations and the phylogeny of the green algae. Biosystems.

[5] Lewis LA, McCourt RM (2004). Green algae and the origin of land plants. Am J Bot.

[6] Pröschold T, Leliaert F: **Systematics of the Green Algae: Conflict of Classic and Modern Approaches.** In *Unravelling the Algae.*: CRC Press; 2007:123–153.

[7] Friedl T, Rybalka N, Luttge U, Beyschlag W, Budel B, Francis D (2012). Systematics of the Green Algae: A Brief Introduction to the Current Status. Progress in Botany 73.

[8] Viprey M, Guillou L, Ferreol M, Vaulot D (2008). Wide genetic diversity of picoplanktonic green algae (Chloroplastida) in the Mediterranean Sea uncovered by a phylum-biased PCR approach. Environ Microbiol.

[9] Huss VAR, Frank C, Hartmann EC, Hirmer M, Kloboucek A, Seidel BM, Wenzeler P, Kessler E (1999). Biochemical taxonomy and molecular phylogeny of the genus *Chlorella* sensu lato (Chlorophyta). J Phycol.

[10] Luo W, Pröschold T, Bock C, Krienitz L (2010). Generic concept in *Chlorella*-related coccoid green algae (Chlorophyta, Trebouxiophyceae). Plant Biology (Stuttgart).

[11] Philippe H, Telford MJ (2006). Large-scale sequencing and the new animal phylogeny. Trends Ecol Evol.

[12] Lemieux C, Otis C, Turmel M: **Six newly sequenced chloroplast genomes from prasinophyte green algae provide insights into the relationships among prasinophyte lineages and the diversity of streamlined genome architecture in picoplanktonic species.** *BMC Genomics* 2014, Accepted for publication on 25 September 2014. 10.1186/1471-2164-15-857 PMC4194372 25281016

[13] Turmel M, Gagnon MC, O’Kelly CJ, Otis C, Lemieux C (2009). The chloroplast genomes of the green algae *Pyramimonas, Monomastix*, and *Pycnococcus* shed new light on the evolutionary history of prasinophytes and the origin of the secondary chloroplasts of euglenids. Mol Biol Evol.

[14] Civan P, Foster PG, Embley MT, Seneca A, Cox CJ (2014). Analyses of charophyte chloroplast genomes help characterize the ancestral chloroplast genome of land plants. Genome Biol Evol.

[15] Lemieux C, Otis C, Turmel M (2007). A clade uniting the green algae *Mesostigma viride* and *Chlorokybus atmophyticus* represents the deepest branch of the Streptophyta in chloroplast genome-based phylogenies. BMC Biol.

[16] Turmel M, Otis C, Lemieux C (2006). The chloroplast genome sequence of *Chara vulgaris* sheds new light into the closest green algal relatives of land plants. Mol Biol Evol.

[17] Turmel M, Pombert JF, Charlebois P, Otis C, Lemieux C (2007). The green algal ancestry of land plants as revealed by the chloroplast genome. Int J Plant Sci.

[18] Zhong B, Xi Z, Goremykin VV, Fong R, McLenachan PA, Novis PM, Davis CC, Penny D (2014). Streptophyte algae and the origin of land plants revisited using heterogeneous models with three new algal chloroplast genomes. Mol Biol Evol.

[19] Brouard JS, Otis C, Lemieux C, Turmel M (2010). The exceptionally large chloroplast genome of the green alga *Floydiella terrestris* illuminates the evolutionary history of the Chlorophyceae. Genome Biol Evol.

[20] Turmel M, Brouard JS, Gagnon C, Otis C, Lemieux C (2008). Deep division in the Chlorophyceae (Chlorophyta) revealed by chloroplast phylogenomic analyses. J Phycol.

[21] Cocquyt E, Verbruggen H, Leliaert F, De Clerck O (2010). Evolution and cytological diversification of the green seaweeds (Ulvophyceae). Mol Biol Evol.

[22] Fučíková K, Lewis PO, Lewis LA (2014). Putting incertae sedis taxa in their place: a proposal for ten new families and three new genera in Sphaeropleales (Chlorophyceae, Chlorophyta). J Phycol.

[23] Friedl T (1995). Inferring taxonomic positions and testing genus level assignments in coccoid green lichen algae: a phylogenetic analysis of 18S ribosomal RNA sequences from *Dictyochloropsis reticulata* and from members of the genus *Myrmecia* (Chlorophyta, Trebouxiophyceae cl. nov.). J Phycol.

[24] Friedl T, Büdel B, Nash TI (2008). Photobionts. Lichen Biology.

[25] Pérez-Ortega S, Ríos A, Crespo A, Sancho LG (2010). Symbiotic lifestyle and phylogenetic relationships of the bionts of *Mastodia tessellata* (Ascomycota, *incertae sedis*). Am J Bot.

[26] Pröschold T, Darienko T, Silva PC, Reisser W, Krienitz L (2011). The systematics of *Zoochlorella* revisited employing an integrative approach. Environ Microbiol.

[27] de Koning AP, Keeling PJ (2006). The complete plastid genome sequence of the parasitic green alga Helicosporidium sp. is highly reduced and structured. BMC Biol.

[28] Pombert JF, Blouin NA, Lane C, Boucias D, Keeling PJ (2014). A lack of parasitic reduction in the obligate parasitic green alga *Helicosporidium*. PLoS Genet.

[29] Ueno R, Urano N, Suzuki M (2003). Phylogeny of the non-photosynthetic green micro-algal genus *Prototheca* (Trebouxiophyceae, Chlorophyta) and related taxa inferred from SSU and LSU ribosomal DNA partial sequence data. FEMS Microbiol Lett.

[30] Hannon M, Gimpel J, Tran M, Rasala B, Mayfield S (2010). Biofuels from algae: challenges and potential. Biofuels.

[31] Mata TM, Martins AA, Caetano NS (2010). Microalgae for biodiesel production and other applications: A review. Renew Sust Energ Rev.

[32] Bock C, Luo W, Kusber W-H, Hegewald E, Pazoutova M, Krienitz L (2013). Classification of crucigenoid algae: Phylogenetic position of the reinstated genus *Lemmermannia*, *Tetrastrum* spp. *Crucigenia tetrapedia*, and *C. lauterbornii* (Trebouxiophyceae, Chlorophyta). J Phycol.

[33] Darienko T, Gustavs L, Mudimu O, Menendez CR, Schumann R, Karsten U, Friedl T, Proeschold T (2010). *Chloroidium*, a common terrestrial coccoid green alga previously assigned to *Chlorella* (Trebouxiophyceae, Chlorophyta). Eur J Phycol.

[34] Elias M, Neustupa J, Skaloud P (2008). *Elliptochloris bilobata* var. *corticola* var. nov (Trebouxiophyceae, Chlorophyta), a novel subaerial coccal green alga. Biologia (Bratislava).

[35] Karsten U, Friedl T, Schumann R, Hoyer K, Lembcke S (2005). Mycosporine-like amino acids and phylogenies in green algae: *Prasiola* and its relatives from the Trebouxiophyceae (Chlorophyta). J Phycol.

[36] Krienitz L, Bock C, Luo W, Pröschold T (2010). Polyphyletic origin of the *Dictyosphaerium* morphotype within Chlorellaceae (Trebouxiophyceae). J Phycol.

[37] Neustupa J, Elias M, Skaloud P, Nemcova Y, Sejnohova L (2011). *Xylochloris irregularis* gen. et sp. nov. (Trebouxiophyceae, Chlorophyta), a novel subaerial coccoid green alga. Phycologia.

[38] Neustupa J, Nemcova Y, Vesela J, Steinova J, Skaloud P (2013). *Leptochlorella corticola* gen. et sp. nov. and *Kalinella apyrenoidosa* sp. nov.: two novel *Chlorella*-like green microalgae (Trebouxiophyceae, Chlorophyta) from subaerial habitats. Int J Syst Evol Microbiol.

[39] Sluiman HJ, Guihal C, Mudimu O (2008). Assessing phylogenetic affinities and species delimitations in Klebsormidiales (Streptophyta): Nuclear-encoded rDNA phylogenies and its secondary structure models in *Klebsormidium*, *Hormidiella*, and *Entransia*. J Phycol.

[40] Krienitz L, Bock C (2012). Present state of the systematics of planktonic coccoid green algae of inland waters. Hydrobiologia.

[41] Pröschold T, Bock C, Luo W, Krienitz L (2010). Polyphyletic distribution of bristle formation in Chlorellaceae: *Micractinium*, *Diacanthos*, *Didymogenes* and *Hegewaldia* gen. nov. (Trebouxiophyceae, Chlorophyta). Phycol Res.

[42] Turmel M, Otis C, Lemieux C (2009). The chloroplast genomes of the green algae *Pedinomonas minor*, *Parachlorella kessleri*, and *Oocystis solitaria* reveal a shared ancestry between the Pedinomonadales and Chlorellales. Mol Biol Evol.

[43] **Culture Collection of Algae at the University of Goettingen.** http://www.uni-goettingen.de/en/45175.html.

[44] **The Culture Collection of Algae at The University of Texas at Austin.** http://web.biosci.utexas.edu/utex/default.aspx.

[45] **Provasoli-Guillard National Center for Marine Algae and Microbiota.** https://ncma.bigelow.org.

[46] **Microbial Culture Collection at the National Institute of Environmental Studies.** http://mcc.nies.go.jp.

[47] **Culture Collection of Algae of Charles University in Prague.** http://botany.natur.cuni.cz/algo/caup-list.html.

[48] Sluiman HJ, Blommers PCJ (1990). Ultrastructure and taxonomic position of *Chlorosarcina stigmatica* Deason (Chlorophyceae, Chlorophyta). Arch Protistenkdr.

[49] Cox CJ, Li B, Foster PG, Embley TM, Civan P (2014). Conflicting phylogenies for early land plants are caused by composition biases among synonymous substitutions. Syst Biol.

[50] Li B, Lopes JS, Foster PG, Embley TM, Cox CJ (2014). Compositional biases among synonymous substitutions cause conflict between gene and protein trees for plastid origins. Mol Biol Evol.

[51] Rota-Stabelli O, Lartillot N, Philippe H, Pisani D (2013). Serine codon-usage bias in deep phylogenomics: pancrustacean relationships as a case study. Syst Biol.

[52] Blanquart S, Lartillot N (2008). A site- and time-heterogeneous model of amino acid replacement. Mol Biol Evol.

[53] Foster PG, Hickey DA (1999). Compositional bias may affect both DNA-based and protein-based phylogenetic reconstructions. J Mol Evol.

[54] Cox CJ, Foster PG (2013). A 20-state empirical amino-acid substitution model for green plant chloroplasts. Mol Phylogenet Evol.

[55] Le SQ, Dang CC, Gascuel O (2012). Modeling protein evolution with several amino acid replacement matrices depending on site rates. Mol Biol Evol.

[56] Lartillot N, Brinkmann H, Philippe H (2007). Suppression of long-branch attraction artefacts in the animal phylogeny using a site-heterogeneous model. BMC Evol Biol.

[57] Lartillot N, Philippe H (2004). A Bayesian mixture model for across-site heterogeneities in the amino-acid replacement process. Mol Biol Evol.

[58] Philippe H, Brinkmann H, Copley RR, Moroz LL, Nakano H, Poustka AJ, Wallberg A, Peterson KJ, Telford MJ (2011). Acoelomorph flatworms are deuterostomes related to *Xenoturbella*. Nature.

[59] Philippe H, Brinkmann H, Lavrov DV, Littlewood DTJ, Manuel M, Worheide G, Baurain D (2011). Resolving difficult phylogenetic questions: why more sequences are not enough. PLoS Biol.

[60] Shimodaira H (2002). An approximately unbiased test of phylogenetic tree selection. Syst Biol.

[61] Shimodaira H, Hasegawa M (2001). CONSEL: for assessing the confidence of phylogenetic tree selection. Bioinformatics.

[62] Regier JC, Shultz JW, Zwick A, Hussey A, Ball B, Wetzer R, Martin JW, Cunningham CW (2010). Arthropod relationships revealed by phylogenomic analysis of nuclear protein-coding sequences. Nature.

[63] Lu F, Xu W, Tian C, Wang G, Niu J, Pan G, Hu S (2011). The *Bryopsis hypnoides* plastid genome: multimeric forms and complete nucleotide sequence. PLoS One.

[64] Novis PM, Smissen R, Buckley TR, Gopalakrishnan K, Visnovsky G (2013). Inclusion of chloroplast genes that have undergone expansion misleads phylogenetic reconstruction in the Chlorophyta. Am J Bot.

[65] Škaloud P, Kalina T, Nemjová K, De Clerck O, Leliaert L (2013). Morphology and phylogenetic position of the freshwater green microalgae *Chlorochytrium* (Chlorophyceae) and *Scotinosphaera* (Scotinosphaerales, ord. nov., Ulvophyceae). J Phycol.

[66] Smith DR, Burki F, Yamada T, Grimwood J, Grigoriev IV, Van Etten JL, Keeling PJ (2011). The GC-rich mitochondrial and plastid genomes of the green alga *Coccomyxa* give insight into the evolution of organelle DNA nucleotide landscape. PLoS One.

[67] Zuccarello GC, Price N, Verbruggen H, Leliaert F (2009). Analysis of a plastid multigene data set and the phylogenetic position of the marine macroalga *Caulerpa filiformis* (Chlorophyta). J Phycol.

[68] Holzinger A, Karsten U (2013). Desiccation stress and tolerance in green algae: consequences for ultrastructure, physiological and molecular mechanisms. Front Plant Sci.

[69] Moniz MBJ, Rindi F, Novis PM, Broady PA, Guiry MD (2012). Molecular phylogeny of Antarctic *Prasiola* (Prasiolales, Trebouxiophyceae) reveals extensive cryptic diversity. J Phycol.

[70] Gaysina L, Nemcova Y, Skaloud P, Sevcikova T, Elias M (2013). *Chloropyrula uraliensis* gen. et sp nov (Trebouxiophyceae, Chlorophyta), a new green coccoid alga with a unique ultrastructure, isolated from soil in South Urals. J Syst Evol.

[71] Fučíková K, Lewis PO, Lewis LA: **Widespread desert affiliation of trebouxiophycean algae (Trebouxiophyceae, Chlorophyta) including discovery of three new desert genera.** *Phycol Res* 2014, article published online on 27 August 2014 (DOI: 10.1111/pre.12062).

[72] Krienitz L, Bock C, Nozaki H, Wolf M (2011). SSU rRNA gene phylogeny of morphospecies affiliated to the bioassay alga “*Selenastrum capricornutum*” recovered the polyphyletic origin of crescent-shaped Chlorophyta. J Phycol.

[73] Neustupa J, Nemcova Y, Elias M, Skaloud P (2009). *Kalinella bambusicola* gen. et sp nov (Trebouxiophyceae, Chlorophyta), a novel coccoid *Chlorella*-like subaerial alga from Southeast Asia. Phycol Res.

[74] Somogyi B, Felföldi T, Solymosi K, Makk J, Homonnay ZG, Horváth G, Turcsi E, Böddi B, Márialigeti K, Vörös L (2011). *Chloroparva pannonica* gen. et sp. nov. (Trebouxiophyceae, Chlorophyta) - a new picoplanktonic green alga from a turbid, shallow soda pan. Phycologia.

[75] Somogyi B, Felföldi T, Solymosi K, Flieger K, Márialigeti K, Böddi B, Vörös L (2013). One step closer to eliminating the nomenclatural problems of minute coccoid green algae: *Pseudochloris wilhelmii*, gen. et sp. nov. (Trebouxiophyceae, Chlorophyta). Eur J Phycol.

[76] Marin B (2012). Nested in the Chlorellales or independent class? Phylogeny and classification of the Pedinophyceae (Viridiplantae) revealed by molecular phylogenetic analyses of complete nuclear and plastid-encoded rRNA operons. Protist.

[77] Keller MD, Seluin RC, Claus W, Guillard RRL (1987). Media for the culture of oceanic ultraphytoplankton. J Phycol.

[78] Andersen RA (2005). Algal Culturing Techniques.

[79] Turmel M, Otis C, Lemieux C (2013). Tracing the evolution of streptophyte algae and their mitochondrial genome. Genome Biol Evol.

[80] **Plateforme d’Analyses Génomiques de l’Université Laval.** http://pag.ibis.ulaval.ca/seq/en/index.php.

[81] Margulies M, Egholm M, Altman WE, Attiya S, Bader JS, Bemben LA, Berka J, Braverman MS, Chen YJ, Chen ZT, Dewell SB, Du L, Fierro JM, Gomes XV, Godwin BC, He W, Helgesen S, Ho CH, Irzyk GP, Jando SC, Alenquer MLI, Jarvie TP, Jirage KB, Kim JB, Knight JR, Lanza JR, Leamon JH, Lefkowitz SM, Lei M, Li J (2005). Genome sequencing in microfabricated high-density picolitre reactors. Nature.

[82] Gordon D, Abajian C, Green P (1998). Consed: a graphical tool for sequence finishing. Genome Res.

[83] **Innovation Centre of McGill University and Genome Quebec.** http://www.gqinnovationcenter.com/index.aspx.

[84] Boisvert S, Laviolette F, Corbeil J (2010). Ray: simultaneous assembly of reads from a mix of high-throughput sequencing technologies. J Comput Biol.

[85] Pombert JF, Otis C, Lemieux C, Turmel M (2005). The chloroplast genome sequence of the green alga *Pseudendoclonium akinetum* (Ulvophyceae) reveals unusual structural features and new insights into the branching order of chlorophyte lineages. Mol Biol Evol.

[86] Lagesen K, Hallin P, Rodland EA, Staerfeldt HH, Rognes T, Ussery DW (2007). RNAmmer: consistent and rapid annotation of ribosomal RNA genes. Nucleic Acids Res.

[87] Lowe TM, Eddy SR (1997). tRNAscan-SE: a program for improved detection of transfer RNA genes in genomic sequence. Nucleic Acids Res.

[88] Michel F, Umesono K, Ozeki H (1989). Comparative and functional anatomy of group II catalytic introns - a review. Gene.

[89] Michel F, Westhof E (1990). Modelling of the three-dimensional architecture of group I catalytic introns based on comparative sequence analysis. J Mol Biol.

[90] Edgar RC (2004). MUSCLE: multiple sequence alignment with high accuracy and high throughput. Nucleic Acids Res.

[91] Capella-Gutierrez S, Silla-Martinez JM, Gabaldon T (2009). trimAl: a tool for automated alignment trimming in large-scale phylogenetic analyses. Bioinformatics.

[92] Smith SA, Dunn CW (2008). Phyutility: a phyloinformatics tool for trees, alignments and molecular data. Bioinformatics.

[93] Stamatakis A (2014). RAxML version 8: a tool for phylogenetic analysis and post-analysis of large phylogenies. Bioinformatics.

[94] Lartillot N, Lepage T, Blanquart S (2009). PhyloBayes 3: a Bayesian software package for phylogenetic reconstruction and molecular dating. Bioinformatics.

[95] Rice P, Longden I, Bleasby A (2000). EMBOSS: The European molecular biology open software suite. Trends Genet.

[96] Nenadic O, Greenacre M (2007). Correspondence analysis in R, with two- and three-dimensional graphics: The ca package. J Stat Software.

[97] Castresana J (2000). Selection of conserved blocks from multiple alignments for their use in phylogenetic analysis. Mol Biol Evol.

[98] Maddison WP, Maddison DR: **Mesquite: A Modular System for Evolutionary Analysis. Version 2.75.** In 2011. http://mesquiteproject.org.

[99] Lemieux C, Otis C, Turmel M: **Data from: Chloroplast Phylogenomic Analysis Resolves Deep-Level Relationships Within the Green Algal Class Trebouxiophyceae.** In http://dx.doi.org/10.5061/dryad.q4432. 10.1186/s12862-014-0211-2 PMC4189289 25270575

